# Supplementary material for: Spatiotemporal variability of oxygen concentration in coral reefs of Gorgona Island (Eastern Tropical Pacific) and its effect on the coral Pocillopora capitata
Source: PeerJ. 2023 Jan 26;11:e14586. doi: 10.7717/peerj.14586 (PMC9884479; doi:10.7717/peerj.14586)
Supplement: Supplemental Information 3 [file peerj-11-14586-s003.docx]

**Supplementary material**.

***1. List of the technical reports assessed to extract dissolved oxygen conditions in reefs of Gorgona island, Colombian Pacific.***

Giraldo, A., B. Valencia, D. Ramírez, J. Jaramillo, D. Lozano. 2008. Condiciones oceanográficas durante noviembre 2008 en el PNN Gorgona. Informe técnico. Grupo de Investigación en Ecología de Arrecifes Coralinos. Facultad de Ciencias, Universidad del Valle.

Giraldo, A. 2010. Condiciones fisico-quimicas del ambiente pelágico del PNN Gorgona durante noviembre 2009. Informe técnico Final. Expedición Gorgona convenio Cooperación Univalle – PNN Gorgona. Grupo de Investigación en Ciencias Oceanográficas, Facultad de Ciencias, Universidad del Valle. 14 p.

Giraldo, A. & Valencia B. 2013. Plancton y condiciones oceanográficas en el PNN Gorgona, Informe técnico de las campañas oceanográficas: septiembre de 2011, marzo de 2012 y septiembre de 2012. Grupo de Investigación en Ciencias Oceanográficas, Departamento de Biología, Facultad de Ciencias Naturales y Exactas, Universidad del Valle. A.A. 25360, Cali - Colombia. 18p.

Giraldo, A. & Valencia, B. 2013. Monitoreo del ambiente pelágico del PNN Gorgona: marzo 2013. Informe Técnico producto No. 8 del proyecto de investigación “Monitoreo de los valores objeto de conservación priorizados para las áreas protegidas Gorgona y Utría adscritas a la Dirección Territorial Pacífico”. Universidad del Valle, Facultad de Ciencias Naturales y Exactas, Departamento de Biología. Grupo de Investigación en Ciencias Oceanográficas. Cali, Colombia. 15 p.

Valencia, B. & A. Giraldo. 2015. Condiciones oceanográficas en Isla Gorgona: relación entre los indicadores biológicos del ambiente pelágico (biomasa del zooplancton y larvas de peces) y las condiciones físico-quimicas de la columna de agua. Informe técnico. Grupo de Investigación en Ciencias Oceanográficas, Departamento de Biología, Facultad de Ciencias Naturales y Exactas, Universidad del Valle. Cali, Colombia. 37 p.

Rivera Gómez, M & A. Giraldo.2016. Monitoreo oceanográfico en el ecosistema pelágico del Parque Nacional Natural Gorgona, durante 2016 (Marzo y Septiembre). Universidad del Valle Facultad de Ciencias Departamento de Biología, Grupo de Investigación en Ciencias Oceanográficas. 10p

Rivera Gómez, M & A. Giraldo. 2018. Condiciones oceanográficas en el Parque Nacional Natural Gorgona, durante marzo y septiembre de 2017. Informe final Fundación Calima – Parque Nacional Natural Gorgona. 82p

Giraldo, A., W. Bolívar-García y M. Rivera-Gómez. 2018. Resultado de monitoreo de ambiente pelágico, anfibios – reptiles y mamíferos en el PNN Gorgona durante 2018. Informe técnico final del Convenio de asociación no. 004 de 2018 suscrito entre Parques Nacionales Naturales De Colombia (Direccion Territorial Pacifico) y Fndación para la Investigación de la Biodiversidad y Conservación en el Trópico. Cali. 63p

Giraldo, A. & W. Bolívar-García. 2019. Resultado de monitoreo de ambiente pelágico y anfibios – reptiles en el PNN Gorgona durante 2019. Informe técnico final del Convenio de asociación no. 003 de 2018 suscrito entre Parques Nacionales Naturales De Colombia (Direccion Territorial Pacifico) y Fndación para la Investigación de la Biodiversidad y Conservación en el Trópico. Cali. 44 p.

***2. Statistical analysis to examine the differences between results obtained from the R packages* respR *and* respirometry *to calculate metabolic rate (MO_2_) of Pocillopora capitata.***

| Variable | T-tests; Group 1: respR Group 2: respirometry | | | | | | | | | | | | | |
| --- | --- | --- | --- | --- | --- | --- | --- | --- | --- | --- | --- | --- | --- | --- |
|  | \| Mean respR \| \| --- \| | \| Mean  respirometry \| \| --- \| | \| t-value \| \| --- \| | \| df \| \| --- \| | \| p \| \| --- \| | \| Valid N respR \| \| --- \| | \| Valid N respirometry \| \| --- \| | \| SD. respR \| \| --- \| | \| SD. respirometry \| \| --- \| | \| F-ratio Variances \| \| --- \| | \| p Variances \| \| --- \| | \| Levene F(1,df) \| \| --- \| | \| df Levene \| \| --- \| | \| p Levene \| \| --- \| |
| \| MO2 \| \| --- \| | 0.25 | 0.26 | -0.79 | 12 | 0.43 | 7 | 7 | 0.02 | 0.02 | 1.02 | 0.97 | 0.000983 | 12 | 0.97 |

Normality of residuals: Kolmogorov-Smirnov test with Lilliefors correction, *D* = 0.21, *p* = 0.1

***3. Statistical analysis to examine the differences in results obtained from the* respR *package to calculate the critical oxygen tension (Pcrit) of Pocillopora capitata using different methods and different widths within methods.***

| Effect | Nested Anova | | | | | | | |
| --- | --- | --- | --- | --- | --- | --- | --- | --- |
|  | \| SS \| \| --- \| | \| df \| \| --- \| | \| MS \| \| --- \| | \| F \| \| --- \| | \| p \| \| --- \| | \| Partial eta-squared \| \| --- \| | \| Non-centrality \| \| --- \| | \| Observed power (alpha=0.05) \| \| --- \| |
| \| Intercept \| \| --- \| | 358.35 | 1 | 358.35 | 437.71 | 0.00 | 0.94 | 437.71 | 1.000000 |
| \| method \| \| --- \| | 0.03 | 1 | 0.03 | 0.04 | 0.84 | 0.001 | 0.04 | 0.05 |
| \| width(method) \| \| --- \| | 4.73 | 2 | 2.36 | 2.89 | 0.07 | 0.19 | 5.78 | 0.51 |
| \| Error \| \| --- \| | 19.64 | 24 | 0.81 |  |  |  |  |  |

Assumptions: homogeneity of variance [Cochran test, C = 0.3, *df* = 3, *p* = 0.6], normal distribution of residual [Kolmogorov-Smirnov test with Lilliefors correction, *D* = 1.19, *p* < 0.2]).

***4. Statistical analysis to examine the differences in results obtained from the* respirometry *package to calculate the critical oxygen tension (Pcrit) of Pocillopora capitata with three different methods. A) ANOVA table, B) Results of Tukey multiple comparisons test.***

A)

| Effect | One-way Anova | | | | |
| --- | --- | --- | --- | --- | --- |
|  | \| SS \| \| --- \| | \| df \| \| --- \| | \| MS \| \| --- \| | \| F \| \| --- \| | \| p \| \| --- \| |
| \| Intercept \| \| --- \| | 201.02 | 1 | 201.0258 | 160.7783 | 0.000 |
| \| method \| \| --- \| | 23.53 | 2 | 11.7663 | 9.4106 | 0.001 |
| \| Error \| \| --- \| | 22.50 | 18 | 1.2503 |  |  |

Assumptions: homogeneity of variance [Cochran test, C = 0.5, *df* = 2, *p* = 0.3]; normal distribution of residuals [*D* = 0.14, *p* > 0.2]

B)

| Cell No. | Tukey HSD test; Between MS error = 1.2503, df = 18.0 | | | |
| --- | --- | --- | --- | --- |
|  | \| method \| \| --- \| | \| {1} 1.5980 \| \| --- \| | \| {2} 3.7916 \| \| --- \| | \| {3} 3.8923 \| \| --- \| |
| \| 1 \| \| --- \| | alpha |  |  |  |
| \| 2 \| \| --- \| | Breakpoint | 0.004 |  |  |
| \| 3 \| \| --- \| | NLR | 0.003 | 0.984 |  |

***5. Statistical analysis to examine the differences in results obtained from the* respirometry *and* respR *packages (and the respective methods that each one employs) to calculate the critical oxygen tension (Pcrit) of Pocillopora capitata.***

| Effect | Nested Anova | | | | | | | |
| --- | --- | --- | --- | --- | --- | --- | --- | --- |
|  | \| SS \| \| --- \| | \| df \| \| --- \| | \| MS \| \| --- \| | \| F \| \| --- \| | \| p \| \| --- \| | \| Partial  eta-squared \| \| --- \| | \| Non-centrality \| \| --- \| | \| Observed power  (alpha=0.05) \| \| --- \| |
| \| Intercept \| \| --- \| | 519.03 | 1 | 519.03 | 487.03 | 0.0000 | 0.91 | 487.03 | 1.00 |
| \| package \| \| --- \| | 2.80 | 1 | 2.80 | 2.63 | 0.1118 | 0.05 | 2.63 | 0.35 |
| \| method(package) \| \| --- \| | 23.56 | 3 | 7.85 | 7.37 | 0.0004 | 0.33 | 22.11 | 0.97 |
| \| Error \| \| --- \| | 46.89 | 44 | 1.06 |  |  |  |  |  |

| Cell No. | Tukey HSD test; variable Pcrit. Approximate Probabilities for  Post Hoc Tests Error: Between MSE = 1.0657, df = 44.0 | | | | | | |
| --- | --- | --- | --- | --- | --- | --- | --- |
|  | \| package \| \| --- \| | \| method \| \| --- \| | \| {1} 1.59 \| \| --- \| | \| {2} 3.79 \| \| --- \| | \| {3} 389 \| \| --- \| | \| {4} 3.54 \| \| --- \| | \| {5} 3.61 \| \| --- \| |
| \| 1 \| \| --- \| | respirometry | alpha |  |  |  |  |  |
| \| 2 \| \| --- \| | respirometry | Breakpoint | 0.002 |  |  |  |  |
| \| 3 \| \| --- \| | respirometry | NLR | 0.001 | 0.99 |  |  |  |
| \| 4 \| \| --- \| | respR | broken stick | 0.001 | 0.98 | 0.94 |  |  |
| \| 5 \| \| --- \| | respR | segmented | 0.001 | 0.99 | 0.97 | 0,99 |  |

***6. Chlorophyll-a in reefs of Gorgona island during the upwelling and non-upwelling seasons.***

| **Reef** | **Chlorophyll a**  **(mg m^3^)** | **year** | **season** | **Technical report**  **(supplementary material 1)** |
| --- | --- | --- | --- | --- |
| ventana | 0.06 | 2009 | Non-Upwelling | Giraldo 2010 |
| playa blanca | 0.12 | 2009 | Non-Upwelling | Giraldo 2010 |
| planchon | 0.06 | 2009 | Non-Upwelling | Giraldo 2010 |
| remanso | 0.18 | 2009 | Non-Upwelling | Giraldo 2010 |
| camaronera | 0.06 | 2009 | Non-Upwelling | Giraldo 2010 |
| ventana | 5 | 2012 | Upwelling | Giraldo y Valencia 2013 |
| playa blanca | 3 | 2012 | Upwelling | Giraldo y Valencia 2013 |
| planchon | 5 | 2012 | Upwelling | Giraldo y Valencia 2013 |
| remanso | 4 | 2012 | Upwelling | Giraldo y Valencia 2013 |
| camaronera | 3 | 2012 | Upwelling | Giraldo y Valencia 2013 |
| ventana | 5 | 2012 | Upwelling | Giraldo y Valencia 2013 |
| playa blanca | 4.5 | 2012 | Upwelling | Giraldo y Valencia 2013 |
| planchon | 6 | 2012 | Upwelling | Giraldo y Valencia 2013 |
| remanso | 5 | 2012 | Upwelling | Giraldo y Valencia 2013 |
| camaronera | 5 | 2012 | Upwelling | Giraldo y Valencia 2013 |
| ventana | 2 | 2012 | Non-Upwelling | Giraldo y Valencia 2013 |
| playa blanca | 2 | 2012 | Non-Upwelling | Giraldo y Valencia 2013 |
| planchon | 2 | 2012 | Non-Upwelling | Giraldo y Valencia 2013 |
| remanso | 2 | 2012 | Non-Upwelling | Giraldo y Valencia 2013 |
| camaronera | 3.5 | 2012 | Non-Upwelling | Giraldo y Valencia 2013 |
| ventana | 2 | 2012 | Non-Upwelling | Giraldo y Valencia 2013 |
| playa blanca | 2 | 2012 | Non-Upwelling | Giraldo y Valencia 2013 |
| planchon | 2 | 2012 | Non-Upwelling | Giraldo y Valencia 2013 |
| remanso | 3 | 2012 | Non-Upwelling | Giraldo y Valencia 2013 |
| camaronera | 2 | 2012 | Non-Upwelling | Giraldo y Valencia 2013 |
| ventana | 4.5 | 2017 | Upwelling | Rivera-Gomez & Giraldo 2018 |
| playa blanca | 6.5 | 2017 | Upwelling | Rivera-Gomez & Giraldo 2018 |
| planchon | 6.5 | 2017 | Upwelling | Rivera-Gomez & Giraldo 2018 |
| remanso | 6.5 | 2017 | Upwelling | Rivera-Gomez & Giraldo 2018 |
| camaronera | 4.5 | 2017 | Upwelling | Rivera-Gomez & Giraldo 2018 |
| ventana | 6.5 | 2017 | Upwelling | Rivera-Gomez & Giraldo 2018 |
| playa blanca | 7.5 | 2017 | Upwelling | Rivera-Gomez & Giraldo 2018 |
| planchon | 6.5 | 2017 | Upwelling | Rivera-Gomez & Giraldo 2018 |
| remanso | 6.5 | 2017 | Upwelling | Rivera-Gomez & Giraldo 2018 |
| camaronera | 7.5 | 2017 | Upwelling | Rivera-Gomez & Giraldo 2018 |
| ventana | 4.5 | 2017 | Non-Upwelling | Rivera-Gomez & Giraldo 2018 |
| playa blanca | 4.5 | 2017 | Non-Upwelling | Rivera-Gomez & Giraldo 2018 |
| planchon | 4.5 | 2017 | Non-Upwelling | Rivera-Gomez & Giraldo 2018 |
| remanso | 6.5 | 2017 | Non-Upwelling | Rivera-Gomez & Giraldo 2018 |
| camaronera | 6.5 | 2017 | Non-Upwelling | Rivera-Gomez & Giraldo 2018 |
| ventana | 5.3 | 2017 | Non-Upwelling | Rivera-Gomez & Giraldo 2018 |
| playa blanca | 5.3 | 2017 | Non-Upwelling | Rivera-Gomez & Giraldo 2018 |
| planchon | 5.3 | 2017 | Non-Upwelling | Rivera-Gomez & Giraldo 2018 |
| remanso | 5.3 | 2017 | Non-Upwelling | Rivera-Gomez & Giraldo 2018 |
| camaronera | 5.3 | 2017 | Non-Upwelling | Rivera-Gomez & Giraldo 2018 |
| ventana | 4.9 | 2018 | Upwelling | Giraldo et al 2018 |
| playa blanca | 7.7 | 2018 | Upwelling | Giraldo et al 2018 |
| planchon | 7.7 | 2018 | Upwelling | Giraldo et al 2018 |
| remanso | 6.1 | 2018 | Upwelling | Giraldo et al 2018 |
| camaronera | 3.7 | 2018 | Upwelling | Giraldo et al 2018 |
| ventana | 5.7 | 2018 | Upwelling | Giraldo et al 2018 |
| playa blanca | 7.7 | 2018 | Upwelling | Giraldo et al 2018 |
| planchon | 7.7 | 2018 | Upwelling | Giraldo et al 2018 |
| remanso | 7.7 | 2018 | Upwelling | Giraldo et al 2018 |
| camaronera | 5.7 | 2018 | Upwelling | Giraldo et al 2018 |
| ventana | 3.7 | 2018 | Non-Upwelling | Giraldo et al 2018 |
| playa blanca | 2.5 | 2018 | Non-Upwelling | Giraldo et al 2018 |
| planchon | 3.7 | 2018 | Non-Upwelling | Giraldo et al 2018 |
| remanso | 3.7 | 2018 | Non-Upwelling | Giraldo et al 2018 |
| camaronera | 3.7 | 2018 | Non-Upwelling | Giraldo et al 2018 |
| camaronera | 3.7 | 2018 | Non-Upwelling | Giraldo et al 2018 |
| ventana | 4.9 | 2018 | Non-Upwelling | Giraldo et al 2018 |
| playa blanca | 3.7 | 2018 | Non-Upwelling | Giraldo et al 2018 |
| planchon | 4.9 | 2018 | Non-Upwelling | Giraldo et al 2018 |
| remanso | 3.7 | 2018 | Non-Upwelling | Giraldo et al 2018 |
